# Electronic Eye Based on RGB Analysis for the Identification of Tequilas

**DOI:** 10.3390/bios11030068

**Published:** 2021-03-02

**Authors:** Anais Gómez, Diana Bueno, Juan Manuel Gutiérrez

**Affiliations:** Bioelectronics Section, Department of Electrical Engineering, CINVESTAV-IPN, Mexico City 07360, Mexico; arochag@cinvestav.mx (A.G.); dbuenoh@ipn.mx (D.B.)

**Keywords:** biologically inspired, electronic eye, optical methods, RGB analysis, tequila

## Abstract

The present work reports the development of a biologically inspired analytical system known as Electronic Eye (EE), capable of qualitatively discriminating different tequila categories. The reported system is a low-cost and portable instrumentation based on a Raspberry Pi single-board computer and an 8 Megapixel CMOS image sensor, which allow the collection of images of Silver, Aged, and Extra-aged tequila samples. Image processing is performed mimicking the trichromatic theory of color vision using an analysis of Red, Green, and Blue components (RGB) for each image’s pixel. Consequently, RGB absorbances of images were evaluated and preprocessed, employing Principal Component Analysis (PCA) to visualize data clustering. The resulting PCA scores were modeled with a Linear Discriminant Analysis (LDA) that accomplished the qualitative classification of tequilas. A Leave-One-Out Cross-Validation (LOOCV) procedure was performed to evaluate classifiers’ performance. The proposed system allowed the identification of real tequila samples achieving an overall classification rate of 90.02%, average sensitivity, and specificity of 0.90 and 0.96, respectively, while Cohen’s kappa coefficient was 0.87. In this case, the EE has demonstrated a favorable capability to correctly discriminated and classified the different tequila samples according to their categories.

## 1. Introduction

Tequila is the traditional Mexican spirit made with agave *tequilana weber* (blue variety), which is grown in five states of Mexico, namely: Guanajuato, Michoacán, Nayarit, Tamaulipas, and Jalisco. Those geographical regions are established in the Protected Designation of Origin (PDO) [1], which guarantees both the manufacturing procedures and the quality necessary to comply with the strict export specifications from the United States [2] and the European Union [3].

Three main categories of tequila are recognized. The first category is Silver tequila. It is obtained directly from the distillation process without additives; it has a transparent appearance, not necessarily colorless proper to an unaged tequila. The second category is called Aged tequila, which means that the tequila has been aged at least two months using oak casks. This process produces a mellowed product with rich color and flavor. Finally, the third category is known as Extra-aged. This tequila is considered more sophisticated because it has been aged for at least one year in wood or oak recipients with V ≤ 600 L, which has enhanced its flavor with predominant woody notes in its color and aroma [1].

These spirits, whose world consumption ranks fourth after whiskey, vodka, and rum, have a significant presence in more than 120 countries, representing sales of more than 200 million liters per year [4]. Hence, quality control is increasingly important to know, characterize, and monitor its aging process, alcoholic content, and volatile composition that define each kind of tequila’s flavor, color, and characteristic aroma.

Nowadays, several tests are carried out in the laboratory to analyze tequila, most of them performing conventional analytical methods such as UV–Vis spectrophotometry [5], Raman spectroscopy [6], Gas Chromatography-Mass Spectrometry (GC-MS) [7], High-Performance Liquid Chromatography (HPLC) [8], Surface Plasmon Resonance (SPR) [9], and electrochemical analysis [10]. Nonetheless, despite their reliability and accuracy, they have several flaws related to long protocols demanding an analysis period from hours to days to carry out the tests, expensive equipment, and the need for technically qualified personnel, without forgetting that their use is confined to special installations with no online quality control possibilities.

Hence, there is an urgent need for fast, inexpensive, portable, and effective alternatives that achieve reliable, non-destructive analytical measurements. Today, biologically inspired analytical systems are beginning to play an important role in the food industry [11,12]. These technologies, sometimes defined as artificial senses, have the perspective of evaluating complex composition samples by emulating the human senses to determine their relevant characteristics. Essentially, the electronic eye has been designed to mimic human eyesight to analyze color and some other attributes related to the sample’s appearance [13,14]. This task can be performed using computer vision, colorimetric, or spectrophotometric methods [15,16,17].

Usually, an electronic eye is built of technology capable of converting optical images into digital images, subsequently analyzed to identify particularities that allow the characterization of what is being observed, avoiding the subjective interpretation of a person [18]. In this sense, image sensors based on a Charge Coupled Device (CCD) or a Complementary Metal Oxide Semiconductor (CMOS) technology are commonly used. Both types of sensors are essentially made up of metal oxide semiconductors (MOS) distributed in a matrix form, and that each independently constitutes a pixel [19]. Once the images are collected, they are subjected to an image processing phase to improve their quality and extract characteristics requiring specific computational algorithms for their interpretation [20].

Electronic eyes have been proven to have an advantage in foodstuff analysis; their applications cover different food industry areas such as quality control, freshness assessment, shelf-life determination, process monitoring, and authenticity assessment [14]. Although the use of electronic eyes in the analysis of some liquids have widely described during the last two decades, these works were focused on samples such as orange juice [21], coffee [22], virgin olive oils [23], milk [24] and some semi-liquids like honey [25], and yogurt [26]. Only a few studies reported the analysis of alcoholic beverages mainly related to wine [27,28], beer, and vodka [29]. Comparatively, the tequila study still continues using conventional techniques instead of an electronic eye. Most tequila analyses have been focused on the determination of quality characteristics [30], sensory properties related to the distillation process [31], evaluation of volatile compounds [32], the relevance of the aging process [33], authenticity [34,35], and adulterations [36].

This work aims to establish the foundations for using an electronic eye in the qualitative identification of different kinds of tequila. The developed analytical system comprises an acquisition image platform that captures and digitalized images directly from tequila, coupled with a biomimetic processing stage based on the trichromatic theory of color vision [37]. In this context, RGB absorbances of images were evaluated and subsequently discriminated against using a Principal Component Analysis (PCA) and Linear Discriminant Analysis (LDA), with was possible to correctly classify the three main tequila categories coming from the Jalisco state region.

The paper is divided into five sections as follows: Section 1 introduction and state of art, Section 2 describes the materials and methods, including details of tequilas set under study, the electronic eye hardware design, experimental setup, image analysis, and data modeling of the proposed biomimetic vision system; Section 3 reports the experimental results obtained from the proposed RGB component analysis, followed by Section 4 where it discusses and compares the key results achieved with our work compared to those reported with conventional analytical techniques. Finally, Section 5 contains the conclusion together with some directions for future work.

## 2. Materials and Methods

### 2.1. Tequilas under Study

A total of 25 samples of different brands were acquired at the local supermarket, all of them with POD, made with 100% agave and certified by Consejo Regulador del Tequila (CRT, for its acronym in Spanish) to ensure their authenticity. These samples were chosen according to the main described categories and considering that they were made in the state of Jalisco. In this way, the formed set includes 8 Silver, 12 Aged, and 5 Extra-aged tequilas. Table 1 summarizes detailed information about the tequila samples used.

### 2.2. Electronic Eye System

**Figure 1:**
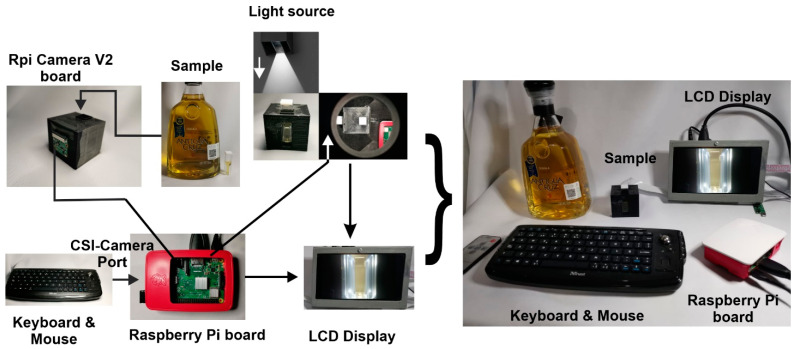
Schematic diagram of the developed Electronic Eye showing different electronic parts assembled and communication interface used.

A single-board computer Raspberry Pi (Model 3B+, Pencoed, Wales, UK) with a Raspbian operating system was chosen as a core for developing the Electronic Eye (EE) prototype. The light source was a white Light-Emitting Diode (LED) 2xLED (Flash Module Huawei LYA-L09, Shenzhen, Guangdong, China), and a camera module (Raspberry Pi Camera V2, Pencoed, Wales, UK) with an 8 Megapixels image sensor (Sony IMX219, Minato, Tokyo, Japan) to perform the image acquisition. It also has a 7-inch Liquid Crystal Display (LCD) (Hilitand hfpq73zx89, Shenzhen, Guangdong, China) that allows interaction with the equipment through a Graphical User Interface (GUI) created in MATLAB® 2020a (MathWorks, Natick, MA, USA). The complete system is managed via Python IDLE 2.7 software, using specific routines programmed by the authors. Figure 1 shows a schematic diagram of the developed EE.

Different EE electronics parts are placed in an enclosure designed in SolidWorks 2019 and printed with a Da Vinci 3D 1.1 printer (Xyzprinting, New Taipei City, Taiwan) to operate as a PC peripheric. The design allows the light source location, camera module, and a disposable plastic UV-cuvette (BRAND, Wertheim, Germany) within a dark chamber. The cuvettes’ filling volume has a range of 1.5 to 3.0 mL, with external dimensions of 4.5 mm × 23 mm that fits into an internal holder of the chamber, allowing it to be located at a fixed distance of 30 mm from the focal plane of the image sensor of the camera. The white LED was positioned in a centered zenith plane to improve accuracy and image acquisition (this position is widely used for samples with flat surfaces) [16]. In this way, white light can propagate from the source, passes from the chamber through the sample held in a cuvette, and reaches the image sensor avoiding possible external interference. At this point, it is possible to acquire the sample’s corresponding digital image. The set of images captured by the EE system were saved automatically in a USB (Universal Serial Bus) device and processed offline employing the GUI designed for this purpose.

### 2.3. Experimental Procedure

After opening each tequila bottle, the spirit was immediately taken. A sample volume of 1 mL of spirit was used directly without pretreatment to fill different UV-cuvettes free of dust and dirt to obtain trustworthy images. Additionally, a cuvette containing the same volume of deionized water was used as a blank solution. All experiments were carried out at room temperature (25 °C). The first measured sample with the EE corresponded to the blank solution to establish a system’s reference signal. Subsequently, the UV-cuvettes containing different samples of tequila were measured one by one. The captured digitized images were recorded and stored using the programmed control software. During the entire experimental stage, it was ensured that the chamber remains closed during the image capture process to avoid the entry of external light and obtain good quality images.

Meanwhile, the white light source stayed on, waiting for the camera module to acquire the image and send it to the Raspberry Pi computer. Each sample was analyzed in triplicate, performing 10 repetitions each time to observe the repeatability and reproducibility of measures. The time to complete the measurement process by the EE system is 10 s.

### 2.4. Image Analysis

Digital images were obtained after placing a UV-cuvette with tequila sample in the lab-made EE system described above. In all cases, the camera settings used in our experiments were fixed (exposure time of 1/16 s, an aperture of f/2, and ISO 100). From the images captured by the EE of each tequila sample and the three categories involved, separate files were saved as a *jpeg* format on the Raspberry Pi memory; the average size per image is 2.7 MB (8 Megapixels resolution, 2592 × 1944 pixels). Although using compressed *jpeg* image format implies a loss of information regarding the *raw* format, some works have reported that the RGB obtained from them contained comparable information to those in large raw files [38,39]. Likewise, *jpeg* files retained the residing color information and allowed ease of handling due to the smaller file size, mainly when some multivariate calibration techniques were used to interpret them [39,40]. In our case, using the *jpeg* format also allowed efficient use of hardware resources (in terms of data storage and computational power requirements), as well as, this image format is closest to the images obtained by the human visual system since they are transformed using color-matching functions [41].

For the image analysis process, it is necessary to perform a preprocessing task that consists of selecting and clipping a region of interest (ROI). The ROI was chosen considering the viewing window of the UV-cuvette. This cropped area of the image and its relative position concerning the sample support is always constant. In this way, the complete set of images were cropped and saved as a separate file with a new dimension size of 1244 × 231 pixels.

Taking into account that digital images are a numeric representation of a two-dimensional collection of data, a digital image contains a fixed number of rows and columns of pixels where each pixel is specified for the red, blue, and green coordinates of a pixel array. This conceptualization of the image is related to the trichromatic theory of color vision based on the work of Maxwell, Young, and Helmholtz [37]. This theory states that there are three types of photoreceptors in the human eye, approximately sensitive to the red, green, and blue region of the spectrum, which are related to the three types of cone cells, generally referred to as L, M, and S (long, medium, and short wavelength sensitivity). These cells are responsible for the perception of colors; analogously, in the RGB color model, the image can be represented by the color’s intensity, which indicates how much red, green, and blue is present in the image [42]. Hence, each component varies from zero to 255 [43]. If all the components are zero, the result is black color. In the opposite case, the result is a white color.

**Figure 2:**
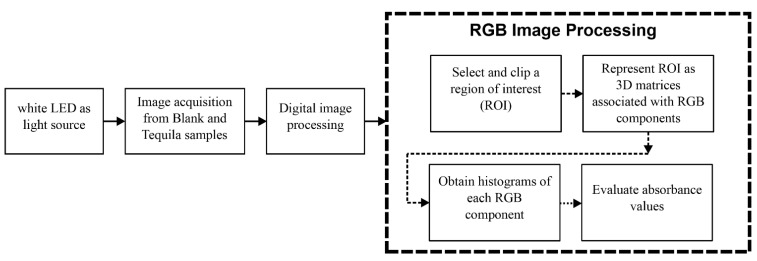
A generalized block diagram of acquisition and identification of image processing performed by the Electronic Eye.

In the same way, considering that the obtained images are true-color images, it is possible to represent them as 3D matrices associated with RGB components. Making it possible to observe its tonal distribution through a histogram and evaluate its corresponding absorbance [44]. The critical steps followed for the EE acquisition and elaboration of RGB images’ regions are illustrated in Figure 2.

The corresponding absorbances associated with the RGB components for the available image set were evaluated using the Lambert–Beer law. This law expresses the proportional relationship between the absorbance and the concentration of certain compounds present in the sample under analysis. The equation representing this law is a crucial element in evaluating the absorbance of a sample [45].

(1) $$A_{\lambda }=-log\left(\frac{I_{1}}{I_{0}}\right)=\varepsilon bC$$

where $A_{\lambda }$ is the absorbance defined via the incident intensity $I_{0}$ (incident light over the sample) and transmitted intensity $I_{1}$ (transmitted light that comes out of the sample), λ is the wavelength of the source light, C is the concentration of the absorbent sample expressed in *moles* * *L*^−1^, b is the optical path (thickness of the cell), and ε is the molar absorptivity coefficient.

Similarly, it is possible to establish that (1) expresses the proportional relationship between the absorbance and the concentration of certain compounds present in the sample under analysis. Consequently, it was part of the implemented algorithms.

Experimentally, when light continues its path from the sample, passes through the camera lens, and reaches the image sensor, some light intensity is lost. This effect is because once a beam of light passes through the UV-cuvette made of transparent material containing the sample, its intensity varies due to the phenomena of absorbance, reflection, and transmission [46]. Therefore, it is possible to compare the light intensity transmitted by a standard (in our case, obtained by a blank solution) and the interest sample’s light intensity. This procedure allows to obtain an experimental absorbance, as shown below in (2):

(2) $$A_{\lambda experimental}=log\left(\frac{I_{solvent}}{I_{analyte\;solution}}\right)$$

where the experimental absorbance $A_{\lambda experimental}$ is evaluated by $I_{solvent}$ related to the blank solution (in this work it was used deionized water) considered a standard sample, and $I_{analyte\;solution}$ corresponding to each tequila sample to be analyzed.

### 2.5. Data Processing and Modelling

Data image processing and modeling were done using the specific routines written in MATLAB® 2020a by the authors, based on already preprogrammed standard functions using Statistics and Machine Learning Toolbox (v11.7). Before carrying out any data processing and modeling task, it was decided to obtain information on the brightness and tonality characteristics of the acquired images to corroborate the equipment’s optical adjustment. For this purpose, histograms of each RGB component were obtained for each available image. Subsequently, the experimental RGB absorbances were calculated (as described in Section 2.4). These calculated values were used as input for two different analysis methods: Principal Component Analysis (PCA) and Linear Discriminant Analysis (LDA). Considering that LDA is a supervised classification method, classification accuracy was evaluated using a Leave-One-Out Cross-Validation (LOOCV) procedure. This iterative method starts using as a training set all the available observations except one, which is excluded for use as validation.

As is known, PCA is an analysis method that depends on an orthogonal linear transformation, which allows summarizing almost all variance contained in a dataset on a fewer number of directions (PCs) with newer coordinates (scores) [47]. In most cases, PCA analysis allows showing clustering data according to their similarities, so it is possible to build a preliminary recognition model that shows the different classes involved according to the measurements made. Nevertheless, to perform a proper classification task, it is necessary to use a supervised learning approach. In this regard, LDA is one of the most used classification procedures with proved successful in many applications [48]. The idea behind LDA is to find a linear transformation that best discriminates among classes. This method operates maximizing between-class variability relative to within-class variability. In this manner, the classification is performed in the transformed space based on some metrics such as Euclidean distance. However, one of the most typical methods to implement is computing a scattering matrix, which must be non-singular. Nonetheless, this criterion cannot be applied when the matrix is singular. A situation that frequently occurs in applications using image databases for pattern recognition, where the number of measurements of each sample exceeds the number of samples in each class. To tackle this problem, it is possible to implement a two-stage approach based on PCA plus LDA. Considering that both methods project the data into a smaller subspace, PCA focused on finding the PCs that maximize the variance in the data set (without considering the class labels), while LDA finds the components that maximize between-class separation. Detailed information about this improved LDA method can be found in [49,50].

## 3. Results

### 3.1. RGB Image Processing

**Figure 3:**
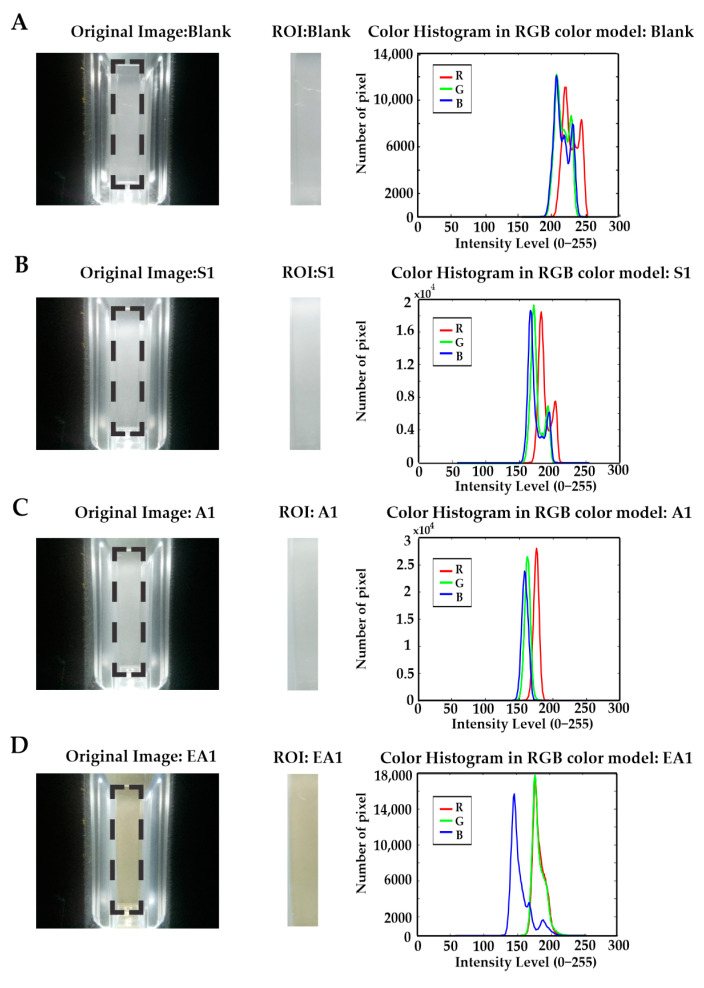
Representative images of (**A**) Blank solution, (**B**) Silver tequila, (**C**) Aged tequila, and (**D**) Extra-aged tequila samples. The image captured by the Electronic Eye (EE), region of interest (ROI) and Red, Green, and Blue (RGB) intensity histogram is noted from left to right.

The experimental phase with the EE allowed capturing a total of 750 tequila images (10 photos for each sample of the 25 tequilas in triplicate). The selected ROI is automatically defined and fixed for all analyzed sample images from these data, as was described in Section 2.4. Figure 3 shows four representative samples and their corresponding captured images for one tequila sample per class plus the blank solution. The black dotted lines within the UV-cuvette image denote ROI selected image area. The histogram visualization shows the presence of reddish, greenish, and blueish pixels in association with the corresponding RGB components of the images. It is possible to show that both the distribution of these color components and their intensity is clearly different for each tequila type. Similarly, it can be assumed that the information captured in the images using fixed camera parameters (exposure time, aperture, and ISO) and under the same lighting conditions is representative to build a classifier model to identify different categories of tequila.

As a reference, color has been one of the important factors in food quality measurement [39]. For this purpose, it is possible to use the RGB model because it is one of the best for detecting color variations of digital images. In this way, the acquired images were organized as a matrix of dimension 30 × 75, where the rows correspond to the total number of repetitions (3 tests with 10 repetitions for each test), and the columns represent the 25 tequila samples analyzed by triplicate. The intensities of RGB components are summarized in Table 2. It also integrated each RGB component’s absorbance and samples’ total absorbance, obtained through Equation (2).

It is possible to establish a relationship between the absorbance and the sample’s content of each image provided by the Electronic Eye. According to the RGB model, an image’s absorbance was calculated about each color component’s average. As shown in Table 2, the average and standard deviation of each color intensity component were obtained together with their related absorbance from different tequila samples’ images.

The Silver tequila sample’s absorbance is 0.0644 ± 0.0034, while for Aged and Extra-aged tequila samples, the average absorbance is 0.0785 ± 0.0024 and 0.0931 ± 0.0019, respectively. The variability presented in the samples can be attributed to the characteristics of each brand’s product, as well as to their particular aging process. Thus, the lowest absorbance values in Silver tequila are associated with its colorless and pure tone.

Depending on the tequila aging process, the tone can be yellowish for Aged tequilas or amber for Extra-aged tequilas. In this manner, while the intensity in the tequila tone increases, the absorbance values also increase.

Related to the RGB components’ intensity, Silver tequila samples showed a prevalence of the three components. However, the Aged tequila samples predominate the red and blue components, whereas the blue component is more present and has the greatest intensity in the Extra-aged tequila samples. These differences have been associated with shades present in samples, despite being the same type of tequila, and these differences depend on the brand.

It is possible to observe that the similarity among obtained measurements for each tequila sample within the same class is minimal since the deviations are in the order of 0.0001–0.0005, demonstrating repeatability in the operativity of the designed EE.

**Figure 4:**
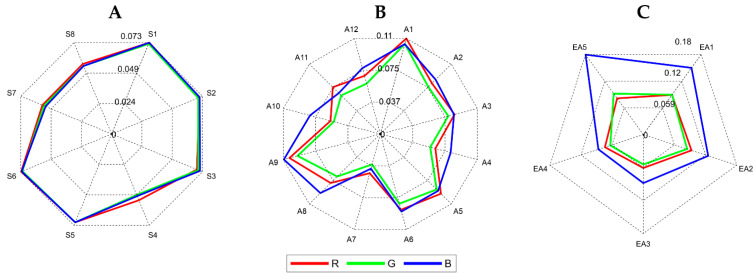
Radar plots for analyzed tequila samples with their respective absorbance values. (**A**) Silver, (**B**) Aged, and (**C**) Extra-aged.

To visualize the behavior of the RGB absorbances of the different tequila samples, radar plots were constructed. Figure 4 shows the RGB average absorbance of the complete set of tequilas grouped in each of the three categories under study. Here it is possible to observe some characteristic fingerprints for each type of tequila related to their optical properties. This evident pattern for each tequila class (i.e., Silver, Aged, and Extra-aged) will help interpret this information by the planned classifier models. The idea behind a pattern recognition process is to recognize the regularities present in data by a computational model that uses machine learning algorithms.

### 3.2. EE Preliminary Recognition Model

**Figure 5:**
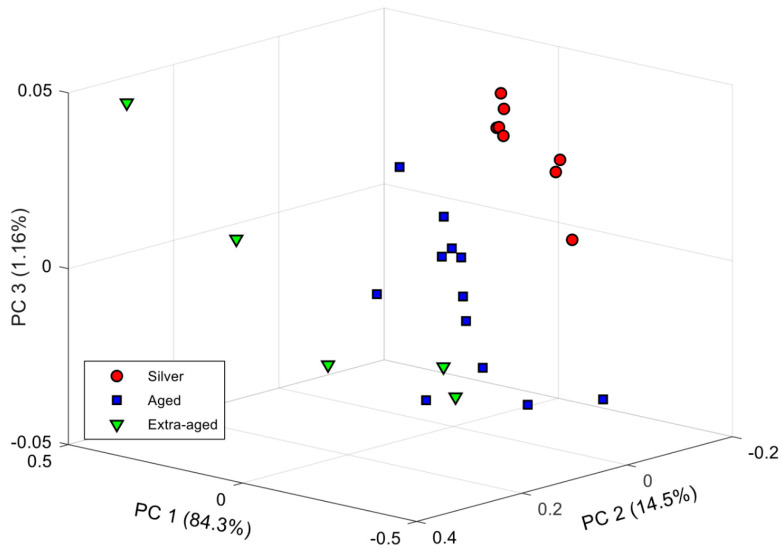
PCA score plot of the three first components obtained after analysis of tequila samples. As can be seen, some clustering is obtained according to different tequila classes.

Before modeling, RGB average absorbances were normalized to an interval of 0 to 1 to reduce illumination effects and for data treatment convenience. Afterward, a PCA analysis was done to build a preliminary recognition model, expecting to observe some sample clustering caused by the own absorbances and tequila class-related. The PCA plot with the three significant PCs is shown in Figure 5. Here the accumulated explained variance was ca. 99.96% with characteristic clusters that partially discriminate the different tequila kinds. That is, most of the Silver tequilas seem to be grouped in the upper right region of the plot, while the Aged tequilas are concentrated in the center, and the Extra-aged ones appear grouped in the left region. However, apart from the marked dispersion of these last two categories of tequilas, there is a clear overlap between some of their samples.

Although the aging mechanisms have been widely studied for different alcoholic beverages such as wine and spirits [51,52], there is still no scientific report that addresses it for tequila. Thus, considering that one of the physicochemical characteristics that are impacted during this process is the color, it is then possible to assume that the absorbances obtained with the electronic eye are also related to the aging of the analyzed tequila samples.

In this sense, the clustering regions observed in the PCA make sense when identifying that samples were grouped within the proper class. On the other hand, each cluster has a relationship with a different aging period. As a result, the dispersion present in the Aged and Extra-aged tequila cluster is clearly related to the aging times that each producer stipulates for their product. On the contrary, in the Silver tequilas cluster, the dispersion is minimal because these tequila samples do not have an aging process.

Thus, it is highly probable that there are tequilas with different aging times within the set of tequila samples analyzed despite belonging to the same category. This may be because each tequila producer must comply with Mexican regulations to respect the minimum aging time. However, they can also establish longer aging periods without violating the standard’s provisions to offer a product with better organoleptic characteristics than their competitions.

For this reason, to confirm these initial identifications seen by PCA, the next step was the use of LDA as a supervised pattern recognition method.

### 3.3. Tequila Categories Discrimination

**Figure 6:**
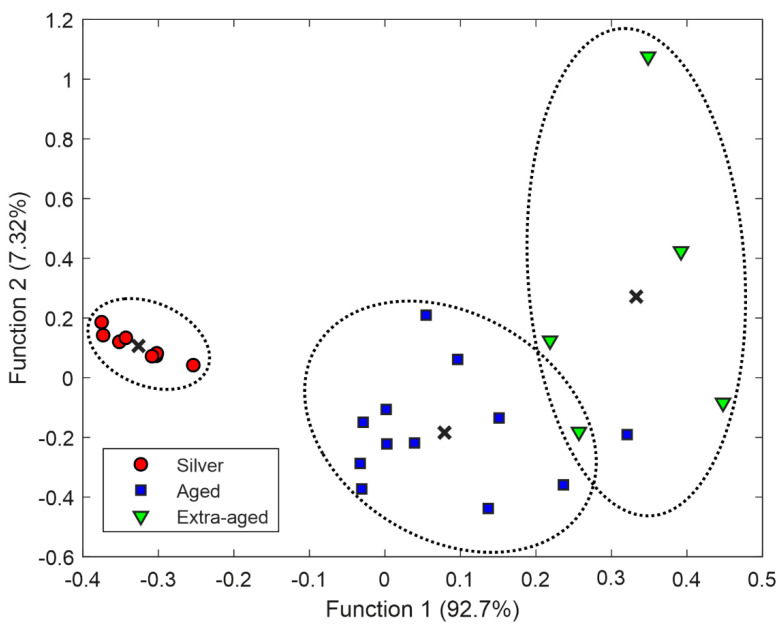
LDA score plot of the obtained functions after analysis of tequila samples, according to their category. Dotted lines represent classification clusters. In addition, the centroid of each class is plotted (x).

Transformed data obtained by PCA were used as input information to perform LDA. Since this is a supervised method, classification success was evaluated using LOOCV. In this scheme, each sample is classified by means of the analysis function derived from the remaining samples (all cases except the case itself). This process was repeated as many times as the number of samples in the data set (i.e., 25 times), leaving out one different sample each time, considering it as a validation sample. With this approach, all samples are used once for validation. As can be observed in Figure 6, clear discrimination between the three categories of tequila was achieved. The clusters in the figure evidence that tequila samples are grouped according to their associated aged process. Although Silver tequilas are clearly grouped on the left region of the plot, the Aged and Extra-aged tequilas have class centroids located in the middle and right regions.

The average classification results obtained from the 25 LDA models built are reported in Table 3. Predictably from the LDA plot, the tequila samples managed to be correctly classified as Silver and Aged, reaching high classification rates (100% and 91.67%, respectively). In contrast, the Extra-aged class did not exceed 78.40% correct classification. The overall classification rate for the three classes was 90.02%. In order to evaluate the efficiency of the modeling, accuracy, precision, sensitivity, and specificity values were also calculated. It is possible to notice that sensitivity averaged for the three classes considered was 0.90, whereas specificity was 0.96.

Many studies have established that the overall classification rate is not the best criterion for measuring classifier performance where there is an imbalance in the number of samples per class [53]. In this direction, to corroborate that the results obtained from the LDA modeling are significant, it is necessary to use another criterion that reflects with more certainty the performance of the classifier in contexts of this imbalance. A well-known alternative measure to the accuracy is Cohen’s kappa coefficient [54]. The fundamental idea for its calculation involves analyzing the differences between the reference data and the incoming data determined by the main diagonal of the confusion matrix, see definition (3).

(3) $$\kappa =\frac{N\sum_{i=1}^{n}m_{i,i}\;-\;\sum_{i=1}^{n}\left(G_{i}C_{i}\right)}{N^{2}\;-\;\sum_{i=1}^{n}\left(G_{i}C_{i}\right)}$$

where i is the class number, N is the total number of classified values compared to truth values, $m_{i,i}$ is the number of values belonging to the truth class i that have also been classified as class i (i.e., values found along the main diagonal of the confusion matrix), $C_{i}$ is the total number of predicted values belonging to class  i, and $G_{i}$ is the total number of truth values belonging to class i.

Thus, kappa is an indicator that acquires values between 0 and 1, the first representing the absolute lack of agreement and the second, total agreement. According to their scheme, a value <0 indicates no agreement, 0–0.20 as slight, 0.21–0.40 as fair, 0.41–0.60 as moderate, 0.61–0.80 as substantial, and 0.81–1 as almost perfect agreement.

In this regard, kappa values were calculated for each of the 25 LDA models built considering the LOOCV process, obtaining an overall mean kappa coefficient of 0.87, which is defined as “perfect agreement”. This finding indicates that this high agreement is related to reliable data. In other words, the RGB absorbances used to identify the tequila samples are representative enough to be modeled. Likewise, although the tequila classes are imbalanced, the LDA models do not privilege the Aged tequila class with the greatest number of samples over the Extra-aged tequila class with the least number of samples.

Additionally, from the obtained results, it is possible to confirm that even using a LOOCV does not produce an over-optimistic approach in the LDA classifiers performance.

## 4. Discussion

The results presented in Section 3 have provided some insight into the developed electronic eye’s capabilities to authenticate the three categories of tequila: Silver (S), Aged (A), and Extra-aged (EA). First, from the preliminary recognition model using PCA, it is important to highlight the close relationship between tequilas’ aging time and their clustering from the RGB absorbance analysis. This same aging effect in tequilas has been observed using more complex analytical methods such as HPLC [8]. This method is responsible for identifying and quantifying low molecular weight phenolic compounds acquired by tequila during the oak barrels’ maturing process. Once characterized, they are related to the mentioned age classifications using analysis of variance (ANOVA) combined with discriminant analysis.

Other works instead deal the authentication of tequila recurring to methods of analysis as GC-MS [34], and UV-Vis [35] coupling some chemometric methods commonly based on LDA, Partial Least Squares Discriminant Analysis (PLS-DA), Multilayer Perceptron Artificial Neuronal Networks (MLP-ANN), and Support Vector Machines (SVM) to name a few. However, although these contributions differ from our study in factors such as the nature of analytical data obtained and the number of tequila samples analyzed, they represent the most recent state-of-the-art in identifying certified tequilas’ three main categories of interest. Added to this, they report performance parameters like sensitivity and specificity of the classifier models they used, which allows direct comparisons with our results. In this way, Table 4 summarizes these parameters’ comparison, including the analytical methods, classification models, and kinds of tequila reported by each research group.

In this way, it is clear that the model adopted in our study using PCA-LDA achieved superior performance in the individualized identification of classes (sensitivity for S = 1.00, A = 0.92, EA = 0.78 and specificity for S = 1.00, A = 0.92, EA = 0.95) than the LDA model (sensitivity for S = 0.66, A = 0.33, EA = 0.66 and specificity for S = 0.75, A = 0.92, EA = 0.73) reported by Ceballos-Magaña et al. [34], and the PLS-DA (sensitivity for S = 0.81, A = 0.71, EA = 1.00 and specificity for S = 0.89, A = 0.88, EA = 0.93) described by Pérez-Caballero et al. [35]. These results are remarkable because, in our study, a linear model was enough to identify tequilas from their RBG absorbances. In contrast, the authors mentioned above needed the use of models with non-linear strategies (e.g., MLP-ANN and SVM) that demand a high computational cost when performing their optimization process to tackle the classification problem properly.

On the other hand, if we compare the results obtained from the non-linear modeling of the PCA-LDA model described in the present work, the overall performance is competitive for the Silver and Aged tequila classes and limited for the Extra-aged class. Finally, the differentiation between non-aged tequilas and those with different maturity levels is closely related to the task of identifying mixed, fake, and adulterated tequilas. Taking into account that adulterations in tequila are also associated with practices such as dilution, the addition of alcohol or some prohibited substances, forbidden aging methods, or blending with lower quality tequila batches, these adulterations are closely related to changes in the UV-vis absorbance and, therefore, in samples’ color [36,39]. Further work will attempt to include these kinds of samples applying the reported image processing procedure in order to find color variations (from RGB absorbances) to identify counterfeit tequilas.

## 5. Conclusions

An electronic eye based on lab-made instrumentation coupled with an image processing stage was developed to build a biologically inspired system capable of distinguishing between different tequila kinds, namely Silver, Aged, and Extra-aged. The system’s repeatability was demonstrated by statistical analysis of the captured images using RGB information. Preliminary analysis employing PCA was relevant to observe data behavior and tequila class clustering mainly related to the aging process. LDA classifiers were built to recognize tequilas through the evaluated RGB absorbances using a LOOCV scheme to identify samples correctly.

Successful discrimination between tequilas was achieved by LDA, obtaining an overall classification rate of 90.02% for the three involved tequila classes mainly associated with their aging process. In the same way, the obtained sensitivity averaged was 0.90, whereas specificity was 0.96. Considering that the analyzed tequila samples are grouped in imbalanced classes, the kappa coefficient was calculated to corroborate that the performance measures were not over-optimistic. In this way, the kappa coefficient mean value was 0.87, which implies that models interpret reliable data without privileging any tequila class after adjustment.

These results show that the developed image analysis strategy based on obtained RGB information of compressed *jpeg* images, together with the PCA-LDA modeling stage, did not hamper the identification of tequilas by retaining enough color information of analyzed samples. Another notable point is that the method presented here agrees with the results reported by some previous studies that employ conventional analytical techniques such as UV-Vis and GC-MS combined with non-linear classification methods. In this sense, the developed electronic eye constitutes a reliable and easy-to-use tool that allows a quick and non-destructive analysis of tequilas to authenticate them according to the three main categories. Lastly, further research may be conducted to identifying fake or mixed tequilas applying the currently reported methodology based on color analysis.

## Figures and Tables

**Table 1:** Tequila samples under study grouped by category.

Type	Brand	Tag	Alcoholic Strength (vol %)
Silver	Hornitos	S1	38
	Orendain	S2	38
	Don Nacho	S3	38
	Corralejo	S4	38
	Dos Coronas	S5	38
	Sombrero Negro	S6	35
	Antigua Cruz	S7	40
	Herradura	S8	46
Aged	Hornitos	A1	38
	Jimador	A2	35
	Jarana	A3	35
	Don Nacho	A4	38
	Cabrito	A5	38
	Antigua Cruz	A6	40
	Don Julio	A7	40
	Dos coronas	A8	38
	Sombrero Negro	A9	38
	Reserva del Señor	A10	35
	Cazadores	A11	38
	Jose Cuervo	A12	38
Extra-aged	Corralejo	EA1	38
	Don Nacho	EA2	38
	Antigua Cruz	EA3	40
	Cazadores	EA4	38
	Hornitos	EA5	35

**Table 2:** Electronic Eye RGB component’s intensities and absorbances values for tequila samples. Furthermore, total absorbance values are reported.

	Average Components of RGB Pixel			Absorbance by Component			$Absorbance_{\lambda }$
Tag	R	G	B	R	G	B	
Blank	255	251 ± 4.0970	253 ± 3.4674	0	0.0026 ± 0.0040	0.0012 ± 0.0027	0.0016 ± 0.0038
S1	215 ± 0.6915	215 ± 1.1861	215 ± 1.0148	0.0729 ± 0.0014	0.0720 ± 0.0024	0.0729 ± 0.0021	0.0726 ± 0.0020
S2	216 ± 1.1059	216 ± 0.8137	216 ± 0.9248	0.0706 ± 0.0023	0.0696 ± 0.0017	0.0708 ± 0.0019	0.0703 ± 0.0019
S3	217 ± 1.7991	216 ± 1.7340	216 ± 1.6046	0.0685 ± 0.0036	0.0696 ± 0.0035	0.0711 ± 0.0033	0.0697 ± 0.0035
S4	225 ± 3.0820	227 ± 3.5519	227 ± 2.8720	0.0529 ± 0.0061	0.0487 ± 0.0070	0.0496 ± 0.0056	0.0504 ± 0.0062
S5	216 ± 1.1427	216 ± 1.1427	216 ± 1.1427	0.0705 ± 0.0023	0.0705 ± 0.0023	0.0705 ± 0.0023	0.0705 ± 0.0023
S6	215 ± 1.0417	215 ± 2.4542	215 ± 1.3113	0.0727 ± 0.0021	0.0717 ± 0.0050	0.0723 ± 0.0027	0.0722 ± 0.0033
S7	223 ± 1.4794	224 ± 2.2733	225 ± 2.8816	0.0556 ± 0.0029	0.0545 ± 0.0045	0.0530 ± 0.0056	0.0544 ± 0.0044
S8	223 ± 3.1220	224 ± 1.8144	224 ± 1.8144	0.0559 ± 0.0062	0.0543 ± 0.0035	0.0543 ± 0.0035	0.0549 ± 0.0044
A1	196 ± 1.3730	199 ± 1.2972	199 ± 1.3730	0.1118 ± 0.0031	0.1055 ± 0.0029	0.1053 ± 0.0030	0.1076 ± 0.0030
A2	211 ± 1.7682	213 ± 1.4794	208 ± 0.8193	0.0812 ± 0.0037	0.0755 ± 0.0031	0.0870 ± 0.0017	0.0813 ± 0.0028
A3	209 ± 0.6215	212 ± 0.7849	209 ± 3.6928	0.0855 ± 0.0013	0.0784 ± 0.0016	0.0850 ± 0.0076	0.0830 ± 0.0036
A4	219 ± 1.2243	222 ± 1.0417	211 ± 1.7207	0.0635 ± 0.0025	0.0576 ± 0.0021	0.0811 ± 0.0036	0.0674 ± 0.0027
A5	204 ± 0.7303	207 ± 0.4498	206 ± 0.6433	0.0955 ± 0.0016	0.0887 ± 0.0010	0.0910 ± 0.0014	0.0917 ± 0.0013
A6	207 ± 0.6915	210 ± 0.6288	206 ± 0.7849	0.0887 ± 0.0015	0.0817 ± 0.0013	0.0908 ± 0.0017	0.0871 ± 0.0015
A7	229 ± 0.6288	234 ± 0.5252	231 ± 0.6065	0.0459 ± 0.0012	0.0356 ± 0.0010	0.0406 ± 0.0012	0.0407 ± 0.0011
A8	212 ± 1.0807	217 ± 0.9965	204 ± 0.8023	0.0784 ± 0.0023	0.0680 ± 0.0020	0.0945 ± 0.0017	0.0803 ± 0.0020
A9	200 ± 1.8889	204 ± 1.2794	197 ± 2.4011	0.1048 ± 0.0042	0.0955 ± 0.0028	0.1113 ± 0.0054	0.1039 ± 0.0041
A10	223 ± 0.8996	224 ± 0.8996	211 ± 3.1397	0.0574 ± 0.0018	0.0537 ± 0.0018	0.0811 ± 0.0067	0.0641 ± 0.0033
A11	214 ± 1.3493	220 ± 0.8137	218 ± 1.3322	0.0748 ± 0.0028	0.0616 ± 0.0016	0.0654 ± 0.0027	0.0673 ± 0.0024
A12	217 ± 0.6288	222 ± 1.2576	213 ± 0.8841	0.0681 ± 0.0013	0.0590 ± 0.0025	0.0771 ± 0.0018	0.0681 ± 0.0019
EA1	207 ± 0.7611	207 ± 1.2015	180 ± 0.4983	0.0884 ± 0.0016	0.0887 ± 0.0026	0.1486 ± 0.0012	0.1086 ± 0.0018
EA2	206 ± 0.7240	210 ± 0.8193	191 ± 0.8683	0.0918 ± 0.0016	0.0836 ± 0.0017	0.1236 ± 0.0020	0.0997 ± 0.0018
EA3	221 ± 0.9248	224 ± 0.7303	208 ± 0.8469	0.0600 ± 0.0019	0.0537 ± 0.0014	0.0872 ± 0.0018	0.0670 ± 0.0086
EA4	215 ± 1.0283	220 ± 0.5509	209 ± 0.8137	0.0731 ± 0.0021	0.0628 ± 0.0011	0.0855 ± 0.0017	0.0738 ± 0.0016
EA5	211 ± 1.2576	206 ± 1.1059	169 ± 0.9685	0.0807 ± 0.0026	0.0912 ± 0.0024	0.1780 ± 0.0025	0.1166±0.0025

**Table 3:** Average classification results of EE for the discrimination of different tequila samples according to expected categories employing PCA-LDA.

Tequila Category	Classification Rate (%)	Accuracy	Precision	Sensitivity	Specificity
Silver	100.00	1.00	1.00	1.00	1.00
Aged	91.67	0.92	0.91	0.92	0.92
Extra-aged	78.40	0.92	0.80	0.78	0.95
Average	90.02	0.94	0.90	0.90	0.96

**Table 4:** Comparison of the current study with representative publications dealing with tequila identification (S = Silver, A = Aged and EA = Extra-aged tequilas).

Reference	Analytical Method	Classification Model	Tequila Category	Sensitivity	Specificity
Ceballos-Magaña et al. [34]	GC-MS	LDA	S	0.66	0.75
			A	0.33	0.92
			EA	0.66	0.73
		MLP-ANN	S	1.00	1.00
			A	0.83	1.00
			EA	1.00	0.93
Pérez-Caballero et al. [35]	UV-Vis	PLS-DA	S	0.81	0.89
			A	0.71	0.88
			EA	1.00	0.93
		SVM	S	1.00	1.00
			A	1.00	0.99
			EA	0.96	1.00
This work	Electronic Eye	PCA-LDA	S	1.00	1.00
			A	0.92	0.92
			EA	0.78	0.95

## Data Availability

Not applicable.

## References

[1] Norma Oficial Mexicana NOM-006-SCFI-2012. http://www.dof.gob.mx/nota_detalle.php?codigo=5282165&fecha=13/12/2012.

[2] Electronic Code of Federal Regulations Title 27, 5.22(g). https://www.ecfr.gov/cgi-bin/text-idx?node=pt27.1.5&rgn=div5.

[3] Council of the European Union Agreement between the European Community and the United Mexican States on the Mutual Recognition and Protection of Designations for Spirit Drinks. https://eur-lex.europa.eu/legal-content/EN/TXT/?uri=CELEX%3A21997A0611%2801%29.

[4] Consejo Regulador del Tequila. https://www.crt.org.mx/index.php/en/pages-2/proteccion-del-tequila-a-nivel-internacional.

[5] Barbosa-García O., Ramos-Ortíz G., Maldonado J.L., Pichardo-Molina J.L., Meneses-Nava M.A., Landgrave J.E.A., Cervantes-Martínez J. (2007). UV–vis absorption spectroscopy and multivariate analysis as a method to discriminate tequila. Spectrochim. Acta Part A Mol. Biomol. Spectrosc..

[6] Frausto-Reyes C., Medina-Gutiérrez C., Sato-Berrú R., Sahagún L.R. (2005). Qualitative study of ethanol content in tequilas by Raman spectroscopy and principal component analysis. Spectrochim. Acta Part A Mol. Biomol. Spectrosc..

[7] De León-Rodríguez A., Escalante-Minakata P., Jiménez-García M.I., Ordoñez-Acevedo L.G., Flores J.L.F., Barba De La Rosa A.P. (2008). Characterization of volatile compounds from ethnic Agave alcoholic beverages by gas chromatography-mass spectrometry. Food Technol. Biotechnol..

[8] Muñoz-Muñoz A.C., Grenier A.C., Gutiérrez-Pulido H., Cervantes-Martínez J. (2008). Development and validation of a High Performance Liquid Chromatography-Diode Array Detection method for the determination of aging markers in tequila. J. Chromatogr. A.

[9] Martínez-López G., Luna-Moreno D., Monzón-Hernández D., Valdivia-Hernández R. (2011). Optical method to differentiate tequilas based on angular modulation surface plasmon resonance. Opt. Lasers Eng..

[10] Oliveira P.R., Lamy-Mendes A.C., Rezende E.I.P., Mangrich A.S., Marcolino Junior L.H., Bergamini M.F. (2015). Electrochemical determination of copper ions in spirit drinks using carbon paste electrode modified with biochar. Food Chem..

[11] Kiani S., Minaei S., Ghasemi-Varnamkhasti M. (2016). Fusion of artificial senses as a robust approach to food quality assessment. J. Food Eng..

[12] Tan J., Xu J. (2020). Applications of electronic nose (e-nose) and electronic tongue (e-tongue) in food quality-related properties determination: A review. Artif. Intell. Agric..

[13] Orlandi G., Calvini R., Pigani L., Foca G., Vasile Simone G., Antonelli A., Ulrici A. (2018). Electronic eye for the prediction of parameters related to grape ripening. Talanta.

[14] Xu C., Zhong J., Wang X. (2019). Electronic eye for food sensory evaluation. Evaluation Technologies for Food Quality.

[15] Wu D., Sun D.W., Kilcast D. (2013). Food colour measurement using computer vision. Instrumental Assessment of Food Sensory Quality.

[16] Wu D., Sun D.-W. (2013). Colour measurements by computer vision for food quality control—A review. Trends Food Sci. Technol..

[17] Gomes J.F.S., Leta F.R. (2012). Applications of computer vision techniques in the agriculture and food industry: A review. Eur. Food Res. Technol..

[18] Ware C., Ware C. (2021). Color. Information Visualization.

[19] Jerram P., Stefanov K., Durini D. (2020). CMOS and CCD image sensors for space applications. High Performance Silicon Imaging.

[20] Patel K.K., Kar A., Jha S.N., Khan M.A. (2012). Machine vision system: A tool for quality inspection of food and agricultural products. J. Food Sci. Technol..

[21] Fernández-Vázquez R., Stinco C.M., Meléndez-Martínez A.J., Heredia F.J., Vicario I.M. (2011). Visual and instrumental evaluation of orange juice color: A consumers’ preference study. J. Sens. Stud..

[22] Buratti S., Benedetti S., Giovanelli G. (2017). Application of electronic senses to characterize espresso coffees brewed with different thermal profiles. Eur. Food Res. Technol..

[23] Apetrei C., Apetrei I.M., Villanueva S., de Saja J.A., Gutierrez-Rosales F., Rodriguez-Mendez M.L. (2010). Combination of an e-nose, an e-tongue and an e-eye for the characterisation of olive oils with different degree of bitterness. Anal. Chim. Acta.

[24] Figueroa A., Caballero-Villalobos J., Angón E., Arias R., Garzón A., Perea J.M. (2020). Using multivariate analysis to explore the relationships between color, composition, hygienic quality, and coagulation of milk from Manchega sheep. J. Dairy Sci..

[25] Shafiee S., Minaei S., Moghaddam-Charkari N., Ghasemi-Varnamkhasti M., Barzegar M. (2013). Potential application of machine vision to honey characterization. Trends Food Sci. Technol..

[26] Abildgaard O.H.A., Kamran F., Dahl A.B., Skytte J.L., Nielsen F.D., Thomsen C.L., Andersen P.E., Larsen R., Frisvad J.R. (2015). Non-Invasive Assessment of Dairy Products Using Spatially Resolved Diffuse Reflectance Spectroscopy. Appl. Spectrosc..

[27] Martin M.L.G.-M., Ji W., Luo R., Hutchings J., Heredia F.J. (2007). Measuring colour appearance of red wines. Food Qual. Prefer..

[28] Ouyang Q., Zhao J., Chen Q. (2014). Instrumental intelligent test of food sensory quality as mimic of human panel test combining multiple cross-perception sensors and data fusion. Anal. Chim. Acta.

[29] Benedetti L.P.d.S., dos Santos V.B., Silva T.A., Filho E.B., Martins V.L., Fatibello-Filho O. (2015). A digital image-based method employing a spot-test for quantification of ethanol in drinks. Anal. Methods.

[30] Villanueva-Rodríguez S.J., Rodríguez-Garay B., Prado-Ramírez R., Gschaedler A., Caballero B., Finglas P.M., Toldrá F. (2016). Tequila: Raw Material, Classification, Process, and Quality Parameters. Encyclopedia of Food and Health.

[31] Prado-Ramírez R., Gonzáles-Alvarez V., Pelayo-Ortiz C., Casillas N., Estarrón M., Gómez-Hernández H.E. (2005). The role of distillation on the quality of tequila. Int. J. Food Sci. Technol..

[32] Martín-del-Campo S.T., López-Ramírez J.E., Estarrón-Espinosa M. (2019). Evolution of volatile compounds during the maturation process of silver tequila in new French oak barrels. LWT.

[33] López-Ramírez J.E., Martín-del-Campo S.T., Escalona-Buendía H., García-Fajardo J.A., Estarrón-Espinosa M. (2013). Physicochemical quality of tequila during barrel maturation. A preliminary study. Cyta-J. Food.

[34] Ceballos-Magaña S.G., de Pablos F., Jurado J.M., Martín M.J., Alcázar Á., Muñiz-Valencia R., Gonzalo-Lumbreras R., Izquierdo-Hornillos R. (2013). Characterisation of tequila according to their major volatile composition using multilayer perceptron neural networks. Food Chem..

[35] Pérez-Caballero G., Andrade J.M., Olmos P., Molina Y., Jiménez I., Durán J.J., Fernandez-Lozano C., Miguel-Cruz F. (2017). Authentication of tequilas using pattern recognition and supervised classification. Trac Trends Anal. Chem..

[36] Contreras U., Barbosa-García O., Pichardo-Molina J.L., Ramos-Ortíz G., Maldonado J.L., Meneses-Nava M.A., Ornelas-Soto N.E., López-de-Alba P.L. (2010). Screening method for identification of adulterate and fake tequilas by using UV–VIS spectroscopy and chemometrics. Food Res. Int..

[37] Wyszecki G., Stiles W. (2000). Color Science: Concepts and Methods, Quantitative Data and Formulae.

[38] Foca G., Masino F., Antonelli A., Ulrici A. (2011). Prediction of compositional and sensory characteristics using RGB digital images and multivariate calibration techniques. Anal. Chim. Acta.

[39] Herrero-Latorre C., Barciela-García J., García-Martín S., Peña-Crecente R.M. (2019). Detection and quantification of adulterations in aged wine using RGB digital images combined with multivariate chemometric techniques. Food Chem. X.

[40] Mutlu A.Y., Kılıç V., Özdemir G.K., Bayram A., Horzum N., Solmaz M.E. (2017). Smartphone-based colorimetric detection via machine learning. Analyst.

[41] Pennebaker W.B., Mitchell J.L. (1993). JPEG: Still Image Data Compression Standard.

[42] Tkalcic M., Tasic J.F. Colour spaces: Perceptual, historical and applicational background. Proceedings of the IEEE Region 8 EUROCON 2003. Computer as a Tool.

[43] Burger W., Burge M.J., Gries D., Schneider F.B. (2016). Color Images. Digital Image Processing: An Algorithmic Introduction Using Java.

[44] Burger W., Burge M.J., Gries D., Schneider F.B. (2016). Histograms and Image Statistics. Digital Image Processing: An Algorithmic Introduction Using Java.

[45] Oldham K.B., Parnis J.M. (2017). Shining light on Beer’s law. ChemTexts.

[46] Corke P. (2017). Light and Color. Robotics, Vision and Control: Fundamental Algorithms In MATLAB.

[47] Jolliffe I.T., Cadima J. (2016). Principal component analysis: A review and recent developments. Philos. Trans. A Math. Phys. Eng. Sci..

[48] Mitteroecker P., Bookstein F. (2011). Linear Discrimination, Ordination, and the Visualization of Selection Gradients in Modern Morphometrics. Evol. Biol..

[49] Swets D.L., Weng J.J. (1996). Using discriminant eigenfeatures for image retrieval. IEEE Trans. Pattern Anal. Mach. Intell..

[50] Belhumeur P.N., Hespanha J.P., Kriegman D.J. (1997). Eigenfaces vs. Fisherfaces: Recognition using class specific linear projection. IEEE Trans. Pattern Anal. Mach. Intell..

[51] Carpena M., Pereira A.G., Prieto M.A., Simal-Gandara J. (2020). Wine aging technology: Fundamental role of wood barrels. Foods.

[52] Delgado-González M.J., García-Moreno M.V., Sánchez-Guillén M.M., García-Barroso C., Guillén-Sánchez D.A. (2021). Colour evolution kinetics study of spirits in their ageing process in wood casks. Food Control.

[53] Sharififar A., Sarmadian F., Malone B.P., Minasny B. (2019). Addressing the issue of digital mapping of soil classes with imbalanced class observations. Geoderma.

[54] Congalton R.G. (1991). A review of assessing the accuracy of classifications of remotely sensed data. Remote Sens. Environ..

